# “Prevention Alone Is Not Enough:” Stakeholders’ Perspectives About School-based Child Sexual Abuse (CSA) Prevention Programs and CSA Research in China

**DOI:** 10.1177/0886260520959630

**Published:** 2020-09-24

**Authors:** Mengyao Lu, Jane Barlow, Franziska Meinck, Yumeng Wu

**Affiliations:** 1 University of Oxford, Oxford, United Kingdom; 2 University of Edinburgh, Edinburgh, United Kingdom; 3 North-West University, Vanderbijlpark, South Africa; 4 Columbia University Irving Medical Center, New York, United States.

**Keywords:** sexual abuse, treatment/intervention, prevention of child abuse

## Abstract

While existing studies have examined the effectiveness of school-based child sexual abuse (CSA) prevention programs in China, there is currently little qualitative evidence on how stakeholders view these programs and research on CSA in China more generally. To address this research gap, the aims of this study were to explore stakeholders’ perspectives on: (a) school-based CSA prevention programs in China; (b) the components of these programs; (c) CSA research in China. Qualitative semi-structured interviews were conducted with 21 participants in Beijing and a county under Lanzhou City, China. Interview transcripts were systematically coded and emerging themes were developed from the codes. An inductive thematic analysis approach was utilized to analyze the interview data. Participants’ perspectives on school-based CSA prevention programs included: (a) recognition of the importance of school-based CSA prevention programs; (b) fear about a possible negative impact on children participating in such programs; (c) assessment that school-based CSA prevention programs alone are not enough to prevent CSA. Components that participants thought needed to be part of Chinese school-based CSA prevention programs were: (a) content regarding online-facilitated CSA; (b) the use of a rights-based approach; and (c) greater parental and community involvement. Participants also identified factors that have both fostered the implementation of CSA research (e.g., the growing awareness of CSA in the central government) and prevented researchers from effectively conducting CSA research: (a) lack of national data; (b) inadequate government support; and (c) barriers to research collaboration among organizations. The findings indicate that while CSA prevention programs are on the whole regarded positively by key stakeholders in China, a number of important concerns were identified. Our study highlighted a number of ways in which future CSA prevention programs and research on CSA could be strengthened in the Chinese context.

## Background

Child sexual abuse (CSA) is a global public health concern and China is not an exception. The most recent meta-analysis on the prevalence of CSA based on questionnaires and face-to-face interviews in China suggested estimates of 9.1% for men and 8.9% for women (Ma, 2018). Evidence from both China and other parts of the world indicates that CSA can lead to significant adverse psychological, behavioral, sexual, and economic consequences (Arriola et al., 2005; Chen et al., 2004; Fang et al., 2015; Luo et al., 2008; Maniglio, 2009; Optimus Study, 2013).

In response to this, efforts have been made to address CSA with different populations, including children, their parents, professionals, and the general public Kenny & Wurtele (2012).  Schools are deemed to be the most promising institution for the delivery of CSA prevention efforts due to their consistent and longitudinal contact with children and their families (Wurtele, 1987). School-based CSA prevention programs are designed to provide students with skills to identify, respond to, and report sexual abuse (Finkelhor, 2009). These prevention programs were first introduced in the United States in the 1970s and were widely implemented across the United States (Berrick & Gilbert, 1991; Finkelhor & Dziuba-Leatherman, 1995). 

A number of systematic reviews have found that participants self-protection skills, for example, recognizing potentially abusive situations, resisting by saying “no” and removing themselves from the potential perpetrator, reporting previous or ongoing CSA incidents to an authority figure, (Wurtele, 1998) increased after participation in the program. These skills were measured through simulation tests (Walsh et al., 2015; Zwi et al., 2007).

The development of school-based CSA prevention programs is still in its infancy in China. Currently, most CSA prevention education content is located within sex education curricula, which are mainly integrated into other subjects such as biology, psychology, and moral education classes (UNESCO & UNFPA, 2018). This integrative model is widely adopted by many schools and it is suggested that little time is allocated to sex education content (UNESCO & UNFPA, 2018). Furthermore, programs focused specifically on CSA prevention have frequently been driven by passionate individual workers or nongovernmental organizations (NGOs) such as *Girls’ Protecting Project* (Girls’ Protecting Fund, 2019). Despite the slow progress, there have been two randomized controlled trials (RCTs; Jin, Chen, Jiang, & Yu, 2017; Lee & Tang, 1998) that examined the effectiveness of school-based CSA prevention programs in China. The results have shown promising results in improving participants’ knowledge and self-protection skills.

However, existing qualitative evidence is limited and is primarily focused on exploring the development of child protection policy and system (Chui & Jordan, 2018), in addition to child protection practices in China (Man et al., 2017; Xu, 2014). The limited research examining stakeholder perspectives is focused mostly on preschool/elementary school teachers’ perceptions of CSA in China. In interviews with 22 preschool and elementary school teachers in Liaoning and Hubei Province regarding their perspectives about CSA in China, Han and Chen (2009) found a paucity of knowledge about CSA among the interviewees: a large number of teachers believed that CSA only includes touching behaviors and was not aware that strangers are not the main perpetrators of CSA. These findings are consistent with a mixed-methods study exploring the implementation of comprehensive sex education in middle schools in China, which suggested that the limited awareness among teachers and the lack of training for teachers are major barriers to teaching sexuality-related content (UNESCO & UNFPA, 2018). The research highlighted the need for school-based CSA curricula and training of preschool and elementary school teachers in China.

Therefore, the aim of the current study was to address the lack of qualitative data and enrich our understanding of stakeholders’ views about existing school-based CSA prevention programs; the components of such programs; and CSA prevention research in China.

## Methods

### Design

A qualitative research design was used, involving individual semi-structured interviews with a range of stakeholders.

### Sampling of Participants

Twenty-one interviewees were recruited between September and November 2019 from universities, NGOs, and inter-governmental organizations in Beijing and an elementary school in a county under Lanzhou city, China. We selected these two sites, one in an urban area and one in a rural area, in order to obtain a wide range of perspectives on the research questions. The inclusion criteria for participants were as follows: (a) individuals who have regular contact with elementary school students in either a personal or a professional capacity (i.e., parents, caregivers, educators who have delivered CSA interventions); or (b) professionals who work in the child protection field (i.e., social workers, representatives of nongovernmental organizations, government officials, child welfare researchers).

### Recruitment

A purposive sampling method was used in which we sought to identify stakeholders with different professional perspectives and varying practical and specific experiences of CSA prevention in China. Purposive sampling allowed for the selection of participants needed to provide information that is particularly relevant to the research questions (Maxwell, 2012). Participants were identified from universities, schools, and organizational websites based on their professional training and expertise. We then employed a snowball sampling method by asking interviewees to recommend additional individuals or organizations to take part in this study. Snowball sampling is considered to be an effective sampling approach to recruit previously “hidden” populations and participants with similar characteristics (Atkinson & Flint, 2001; Parker et al., 2019).

Potential participants were initially contacted through an email, which included details about the purpose of the study and requested them to contact the first author regarding their interest in participating in the study. Where they responded to say that they were interested, the first author would schedule a phone call to discuss their participation further. Where appropriate, the first author then further arranged a mutually agreed upon date and time to complete the recruitment process and conduct the interview.

All the participants were provided with a hard copy of the information sheet, providing detailed information about the purpose of the study, potential risks and benefits, incentives, study procedures, voluntary participation and the right to withdraw, confidentiality, data management, and contact information of the researchers. The first author reviewed the information sheet with participants and provided them with the opportunity to ask questions prior to agreeing to participate in the study. Written consent was obtained prior to participation in the study.

### Ethics Approval

Ethics approval was obtained from the Department of Social Policy and Intervention, University of Oxford and the School of Social Development and Public Policy, Beijing Normal University.

### Data Collection

The study utilized a semi-structured interview schedule. Data collection took place between September 2019 and November 2019. An interview guide with a list of open-ended questions was used to facilitate the stakeholder interviews. Interview questions varied depending on participants’ expertise. For example, interview questions related to CSA intervention programs were put to all the participants, whereas questions related to CSA research, were only put to professionals and researchers. All interviews were conducted in Mandarin Chinese either over the phone or in person in an office or an empty classroom, and audio-recorded with the permission of the interviewee. Interviews ranged from 40 minutes to 80 minutes, with an average length of 60 minutes.

### Data Analysis

An inductive thematic analysis was carried out to analyze the interview transcripts using ATLAS.ti software (Version 8.4.4; Muhr, 2004). All the interviews were transcribed verbatim by the first author immediately after each interview. Upon completion of the transcription process, the first author conducted the initial review of the transcripts and developed a draft codebook, which included definitions of codes, and examples of when to use the code. All authors then reviewed the draft codebook and provided feedback. The first and the last author conducted initial coding using the draft codebook and further revised the codebook based on co-authors’ feedback and the actual coding experience. The first and the last author double-coded all transcripts to ensure intercoder reliability. Discrepancies between the two coders were resolved by discussion. The first and the last author then reviewed the coded content and generated themes. All authors agreed on the final themes and sub-themes. Quotes selected to be presented in this manuscript were translated from Mandarin Chinese to English by the first author and reviewed by the last author, both of whom are native Mandarin Chinese speakers.

## Results

Twenty-five stakeholders were approached to take part in an interview, of whom four declined and 21 participants were interviewed, and their roles are described in Table 1. All participating professionals and university researchers were recruited from universities, NGOs, inter-governmental organizations in Beijing and have experience in either conducting child protection research projects or child protection practice in both rural and urban areas. The school social workers, teachers, and parents were interviewed in an elementary school in a county under Lanzhou city. The student volunteer was based in a child protection organization in Beijing and had rich experiences in teaching CSA prevention lessons.

**Table 1.** Sample Characteristics.

Participants	ID	Number of Participants (*n* = 21)
Professionals		
Nongovernmental organization (NGO) officials	E4, E5, E8	4
University researchers	E1, E3, E6, E7	4
Inter-governmental organization official	E2	1
Practitioners		
School social workers	V1, V2, V3	3
Volunteer	V4	1
Parents	P1, P2, P3, P4, P5, P6	6
Elementary school teachers	T1, T2	2
Total		21
Sex		
Female		19
Male		2

### Views About School-based CSA Intervention Programs

#### Recognition of the need for school-based CSA intervention programs.

The majority of interviewed parents and teachers believed that while the school would provide general safeguarding training related, for example, to fire and water, that there was no training specifically targeting CSA:

There are no specific CSA-related lessons in the school and some (CSA-related) content is integrated into subjects such as moral education and safety training. (T2, elementary school teacher)

While one parent said that her son had learned about saying “no” to suspicious strangers in the safeguarding training, our interviews with the majority of stakeholders suggested that programs targeted at CSA prevention were rarely delivered at schools.

The school provides regular safety trainings including earthquake, fire, and water safety to students. Students would practice the skills that they learned in classrooms. Teachers also teach CSA prevention stuff such as say “no” (to suspicious strangers). (P1, parent)

One teacher also reported the limited CSA-related trainings that they received:

We are not experts in teaching CSA-related content. It feels like we are teaching what we have just learned. We would pay more attention to it (CSA prevention lessons) when the local education authorities put pressure on us. However, most of the time the classes are about general safety trainings. (T1, school teacher)

Despite a lack of CSA-related lessons in the school, parents referred to the importance of school-based CSA intervention programs. All interviewed parents perceived the potential benefits of CSA prevention lessons and wanted their children to participate in such lessons in school. Two factors seemed to underpin this view: (a) their own lack of knowledge with regard to CSA prevention and (b) concerns regarding the potential risks that their children might encounter later in life.

It might be helpful if teachers could teach these classes because as parents, we do not know how to tell them (about this). We did not receive such trainings when we were students. (P1, parent)

Even though it is less dangerous in rural areas, I think that children should learn these skills so they could protect themselves when they grow up and go to schools in bigger cities. (P2, parent)

One NGO official also highlighted the importance of enabling children to protect themselves from CSA through the delivery of school-based CSA prevention programs:

Children’s participation is important in the child protection process. Parents cannot supervise them 24/7 so it is necessary to have these programs in place and teach children self-protection skills in schools. It is adults’ responsibility to protect children; however, it is also our responsibility to teach children to protect themselves. (E5, NGO official)

#### Concerns about school-based CSA prevention programs

##### Concerns about the potential negative impact of such programs.

A number of researchers and professionals were concerned about the possible negative impact of CSA prevention programs. For example, one researcher expressed the view that CSA prevention programs might unintentionally depict sex as dangerous and dirty, and some interviewees also suggested that a comprehensive sex education curriculum was needed:

If the school only allocates one hour to these programs with the sole focus on discussing sexual abuse, I believe this is not comprehensive, it is rather negative. From the child development point of view, positive responses are needed throughout one’s developmental process. I hope that CSA prevention programs could teach children to understand their body from a positive perspective and encourage them to explore their body with confidence and self-esteem. (E3, university researcher)

One school social worker who had delivered CSA lessons also described encountering situations in which parents approached them with concerns regarding the content of the programs:

I think the biggest challenge we are facing is that parents would come to us after reviewing the manual and tell us that they think certain elements in the manual are not age-appropriate for children. (V1, school social worker)

##### Inadequate preparation when delivering CSA prevention programs.

Interviewees who had experience of delivering CSA prevention programs described receiving inadequate preparation. For example, one school social worker described how they had been asked to teach students about CSA prevention after school, without being given any preparation for this role. As a consequence, this member of staff described not knowing how to go about teaching it, and even being unsure with regard to the definition:

It was our first time to do activities (CSA classes) like this, I am still thinking about how to do it.… I am still unclear about the definition of CSA myself, the information that I searched online may not be accurate. (V3, school social worker)

These teachers never received proper sex education while they were in elementary schools and they did not receive relevant training when they were in college either. Also, their parents might not tell them these things. Thus, they do not know how to teach these lessons. (E3, university researcher)

One student volunteer from a child protection NGO reported that the organization provided standardized training prior to teaching the program, but that despite this she experienced difficulties in handling situations where students were disruptive or asked questions that were not covered by the manual:

For lower graders, the biggest challenge is that we don’t have strategies to quieten the class like teachers do. (V4, student volunteer)

Some volunteers could memorize every word in the training manual; however, they feel lost when students ask them questions that are not listed in the manual. (V4, student volunteer)

#### School-based CSA prevention programs alone are not enough.

There was strong consensus among the interviewed professionals and researchers that school-based CSA prevention interventions alone are not enough to prevent CSA given the lack of follow-up services for victims. For example, one school social worker who had delivered CSA prevention programs in elementary schools pointed out that it is challenging to seek support for children who disclosed sexual abuse after the class:

Our class is targeted at preventing CSA and some kids might realize they have been sexually abused after the class. However, we do not have sufficient support for them, especially in rural areas … there is a lack of these kind of resources across the country in general.… There were victims who came to us for help but there was no follow-up on this. (V1, school social worker)

Given the lack of such follow-up services for CSA victims, one NGO official questioned the effectiveness of school-based CSA programs and suggested that school-based CSA prevention approaches were popular because they were easier to implement than support services:

I think that it is necessary to deliver such classes in schools. However, is it the most important step? Or just because it is the easiest approach? Why do people put all the efforts on school settings? I personally doubt the effectiveness of these programs, especially given that there is extremely limited support for CSA victims.… Most of the time NGOs just pick the easiest thing to do. (E4, NGO official)

### Views about the Components of School-based CSA Programs

In order to ascertain the views of interviewees regarding the key components of CSA programs and what else might be needed, a list of the components with definitions was presented to the interviewees (Refer to Table 2).

**Table 2.** Key Components Identified by the First Author (Lu et al., 2020).

Name of the Component	Description
Defining child sexual abuse	Teaching children about what CSA means
Private parts of the body	Teaching children to identify the private parts of their bodies, or to distinguish public and private parts
Safe and unsafe touches	Teaching children to recognize appropriate and inappropriate touches and distinguish between these touches
Self-protection skills	Teaching children self-protection and a range of skills for responding in potentially abusive situations
Children are not the ones to blame	Teaching children that sexual abuse of a child is never the child’s fault
Safe and unsafe situations	Teaching children to recognize safe and unsafe situations
Awareness of personal rights/body ownership	Teaching children their body belongs to them and no one can touch it without their permission
Offender characteristics and approaches	Teaching children that the perpetrators can be someone known or unknown to them and providing information about grooming strategies including gifts and favors
Other harms that can be experienced by children	Teaching children about different forms of harm to children including physical and emotional abuse, witnessing domestic/family violence, or peer victimization such as bullying
Good and bad secrets	Teaching children the difference between good and bad secrets, or to distinguish secrets and surprises
Who to tell	Teaching children about the importance of disclosure and pointing them to appropriate and effective sources of help

#### Component(s) that participants liked and disliked.

There was considerable agreement among interviewees that the identified components were essential in terms of helping children learn about CSA prevention-related skills and knowledge. Specifically, many interviewees believed that the component “offender characteristics and approaches” is very important because children may have misconceptions about potential perpetrators.

I think that it is important to teach children that perpetrators could be someone they have known for a long time. (E2, inter-governmental organization official)

We should tell children that non-strangers could hurt them too. (E4, NGO official)

In terms of components that were disliked, participants stressed that the component “*who to tell*” requires additional consideration in a Chinese context where supporting services for CSA victims are extremely limited. They suggested that disclosure should only be encouraged when supportive services are in place.

Disclosure of CSA does not mean that the victims can get help. Children might be retraumatized if they cannot receive appropriate help after disclosing the CSA experience. (E2, inter-governmental organization official)

*I am not saying that telling children to disclose their abuse is not important, but how should we respond to a child’s disclosure? (E4, NGO official)*

#### Missing components identified by participants.

Three main suggestions for additional components emerged from the interview data: (a) content regarding online-facilitated CSA; (b) the need for a rights-based focus; (c) parental and community engagement.

##### Content regarding online-facilitated CSA.

One professional noted the emerging trend in terms of online-facilitated CSA in China, and discussed the importance of adding content to school-based CSA prevention programs about the risk of abuse on the internet:

but they (CSA programs) can’t cover all the dangerous circumstances. For example, there is a growing trend of online-facilitated CSA currently in China, like forcing children to take or view nude images of themselves.… Unless internet companies remove these photos, but some of them are presented in formats such as cartoons so it is difficult for AI to sense them … so there is a lack of prevention of this kind of CSA form so far. (E2, inter-governmental organization official)

##### Rights-based focus.

The need for a “rights-based” approach was identified as something that should be a key component of Chinese CSA prevention programs as a result of the fact that traditional Chinese culture places a strong emphasis on children’s obeisance to parents and external authority, and to enable children to address the “power imbalance” that occurs with CSA:

Our culture heavily emphasizes authority and children are educated to listen to their parents since they are at a very young age. There is power imbalance here. Therefore, we need to tell children that they have many rights, especially body rights. (E3, university researcher)

The power imbalance between the perpetrator and the CSA victim may lead to severe psychological consequences of the victim.… We need to teach children that they have the power to say “no.” (E6, university researcher)

##### Parental and community involvement.

Parental and community involvement was also identified as being a key element of effective CSA prevention. Interviewees suggested that the prevention of CSA requires involvement of the wider community, including parents. For example, one researcher said:

It is important to provide training to parents and let them know (what we are teaching). Sometimes they do not understand why we are teaching the knowledge to the first graders, we need to let them know that this is good for children’s well-being and overall development. (E3, university researcher)

In addition to educating parents, interviewed researchers suggested the need to educate the wider community in order to ensure that children were not held responsible for preventing child abuse:

Community education is also important in the educational process. Because schools are like bubbles and are not where children live in real life. The biggest problem here is that children might not know how to protect themselves even after receiving the training in the school. As a result, they might blame themselves for not being able to protect themselves. (E7, university researcher)

This interviewee further explained how the community can be protective of children in urban settings:

It has become clearer that community is getting more and more important (in our work). Urban and rural areas are two different concepts: there is a strong sense of “community” in Chinese rural areas, which means individuals are connected with each other and neighbors would help each other if anything happens.… However, the problem with urban areas is that the boundaries are clearly defined, a child can be hidden pretty well in this situation and the neighbors might not even know the child exists. (E7, university researcher)

### Views about CSA Research

#### Facilitators of CSA research implementation.

One official described how the central government has become increasingly aware of the severe child maltreatment cases, many of which are exposed by media, and the negative consequences that child maltreatment can cause, and suggested that this had served as a facilitator of further CSA research:

For example, in 2013, two kids were found starved to death at home in Nanjing because their mother, who is a drug addict, locked them alone at home and left for more than two weeks. After this incident, the government officials realized the severity of child maltreatment and felt pressured by the public. This incident, along with many other child maltreatment cases happened in 2013, improved research in child abuse. (E2, inter-governmental organization official)

#### Barriers to CSA research implementation.

##### Lack of national data.

Researchers and professionals identified the lack of representative data with which to determine the scale of CSA, as being a barrier to addressing CSA and also to the conduct of research on CSA. The need for collecting national-wide data on this matter was repeatedly expressed:

I hope that we could have a large dataset to estimate this issue (CSA prevalence). For example, our research team can only collect data from a limited number of schools, but people would say it does not prove anything because we only include hundreds of students. It is difficult for us to conduct evidence-based research because no national data collection efforts have been made so far. (E3, university researcher)

People do not know where and how to start (collecting data). There is a research collaboration project about (hidden for anonymous purposes) and I was trying to collect data on the number of domestic violence-related incidents. My request was rejected because it was viewed as a sensitive topic. (E4, NGO official)

##### Inadequate government support.

Researchers and professionals also reported that they received limited support from the government when conducting research related to child welfare and child protection more generally. This lack of support was described as being manifest in multiple domains: at the policy level, where the government perceives CSA prevention as being a low priority compared with aging and poverty alleviation, resulting in insufficient funding to the field; and at an individual level, where indifference towards the topic of CSA prevention has led to difficulties for researchers in recruiting government officials for research projects, thereby limiting the researchers’ ability to better understand officials’ perspectives on CSA issues.

Personally, I think the government doesn’t really support scholars’ work. For example, it is very difficult for us to interview government officials. We have to rely on personal connections and go from “the back door,” but we often are still rejected by relevant authorities. (E1, university researcher)

In general, the government’s work is more focused on poverty alleviation and eldercare.… The government always stresses the primary responsibility of the families and the government views families as the “safety nets” of children. So, I don’t think that it (child protection) is the most urgent issue now. (E1, university researcher)

One researcher pointed out that social welfare in China is still at an early stage, and that the lack of government support can be seen across all aspects of social care:

The country is still navigating social service models in all the areas … they don’t know how to connect with each other and there are not many professionals (doing this). In the past, we tended to emphasize “food and clothing” … it was all about meeting basic needs and not enough emphasis on social support. (E6, university researcher)

##### Barriers to research collaboration.

*Lack of awareness regarding existing resources*. Participants described being unaware of what resources for collaboration are available in terms of CSA prevention, and how to initiate such collaboration. They also suggested that it might be beneficial to create platforms such as annual conferences or charity exhibitions to enable social service agencies that conduct work on CSA, to get to know each other’s work. While they believed the government had to take the initiative, some interviewees also expressed concern about this approach because “*things will become difficult when the government enters the scene*.” (V4, student volunteer)

*Failure to view the individual as a whole person*. Most participants shared the view that the government fails to view children as a whole person in relation to CSA prevention and child protection more generally, particularly in terms of the way in which the system works. For example, one researcher, who has considerable experience of working with the government on child protection issues, described the difficulties in terms of the way in which the government works:

The traditional Chinese government structure consists of many vertical lines. For example, the line of Ministry of Health is very smooth from top to bottom and so is the All-China Women’s Federation … but there is no connection horizontal-wise. That is to say, collaboration with wider government initiatives has not been required in the past. However, a child needs to be viewed as a complete person under the framework of Convention on the Right of the Child, and that requires support for different aspects [of their functioning]. This need is overlooked by the central government. (E6, university researcher)

This factor was also perceived to be an obstacle to scaling up CSA prevention programs:

There are laws about mandatory reporting, but what next? How to collect the evidence? When is the best timing to collect the evidence? It is all blank. It is problematic and dangerous to just rely on the police’s judgments.… We hope to break these government barriers because it is difficult to solve children’s issues through just one department. (E4, NGO official)

### Changes Needed

In addition to the above views, participants described changes that they would like to see in the CSA prevention/child protection field in China. Four recommendations were provided and could be broadly divided into two dimensions reflecting the proposed research questions.

#### Changes needed in school-based CSA prevention programs.

##### The need for more evaluation studies of CSA programs.

Many researchers and professionals thought that the conduct of evaluations of school-based CSA programs would be a good opportunity to examine the effectiveness of these programs. For example, one researcher said:

I hope to see more empirical studies evaluating the impact of these curriculums (sex education) on child development. I also hope that we could have a large dataset to explore this.… Unfortunately, our government does not think about doing so. (E3, university researcher)

One NGO official identified the wider culture as being problematic in terms of such evaluation:

I really want to see evaluation studies on these (CSA prevention) programs. I do not think Chinese people trust scientific research. It is not until recently that scientific concepts were adopted to China, our ancestors did not really believe in science [laugh]. (E4, NGO official)

##### Cultural adaptation of child protection systems and CSA prevention programs.

Participants noted that the need for cultural adaptation should be taken into account when developing school-based CSA prevention programs and the child protection system. For example, one NGO official suggested that some of the CSA prevention curricula from western countries may not be suitable to the Chinese cultural context:

There are some Nordic countries are more advanced than us in terms of CSA prevention, but we cannot just simply copy their programs because our culture is more conservative. For example, we would use some references instead of the names of private parts when teaching these lessons. (E5, NGO official)

Another NGO official was interested in learning about how the child protection systems work in western countries and what needs to be done before adopting certain elements:

I would like to see the differences between child protection systems under Chinese and foreign contexts. We can only “copy” their strategies once we have a clear understanding of what works in those systems or programs. However, there are not many scholars conducting research on these topics in China. (E4, NGO official)

#### Changes needed in CSA research in China.

##### Eliminating external risk factors for CSA.

There was a strong consensus among professionals and researchers about the importance of reducing external risk factors for CSA. For example, one researcher highlighted the necessity of educating potential offenders:

Prevention should not just focus on child protection education for children; instead, we should pay attention to people who are not supposed to hurt others.… We should also assume that some of the students who receive such educational trainings will hurt others. We should let them know that it is illegal to do so, and they will be punished for their harmful behaviors. (E6, university researcher)

An NGO official provided recommendations for eliminating external risk factors for CSA. For example, it was suggested that there should be a more sufficient legal definition of CSA to protect children from being sexually abused. The interviewee also described how the lack of an adequate definition of CSA has posed barriers for professionals who work with children:

I think the main challenge is that social workers don’t know how to intervene when investigating CSA cases … [the lack of adequate CSA definition] also poses challenges when it comes to sentencing of the CSA offenders. (E5, NGO official)

Moreover, this interviewee suggested that severe penalties should be in place to deter potential perpetrators from committing CSA acts:

We need harsher punishment for CSA offenders in China. Some countries would arrest people for producing child pornography. We should increase the maximum penalties for CSA offences. (E5, NGO official)

##### Building an integrated child protection system.

Professionals and researchers highlighted the urgent need to establish an integrated child protection system that involved identifying, referring, and supporting CSA victims. Participants argued that one should look at the whole picture when it comes to CSA prevention instead of focusing on one particular point along the prevention treatment continuum. That is to say, *“we should put efforts into preventing, referring, and intervening at different stages of this matter”* (E4, NGO official).

The word “institutionalization” was frequently used by participants when asked how to establish this integrated child protection system. It was suggested that the government should build the platform and take the initiative, rather than social services agencies and NGOs. Professionals with experience in piloting an integrated child protection system suggested that the project they are working on should be scaled up by the central government, and that this would help to form and stabilize the system.

One researcher, who identified challenges while implementing a pilot project on child protection services, perceived the lack of human resources and funding in rural areas as being the major obstacle to delivering and scaling up the service:

In China, the government provides social service primarily through purchasing services from social organizations. The development of social organizations, however, is extremely imbalanced between rural and urban areas. Therefore, we were only able to find child protection professionals from nearby cities to provide services to children in need due to lack of human resources and funding in rural areas. Therefore, it is challenging to link children in need with social services in these areas. (E1, university researcher)

This response echoed the concern raised by a social worker in the rural elementary school, who reported that they *“do not have sufficient support for children who disclose CSA experiences and there is no follow-up regarding their disclosures either.”* (V1, school social worker)

As a result, it was suggested that the central government should protect the implementation of practice by issuing policy mandates and allocating funding to ensure the availability and quality of child protection services in both rural and urban areas and support the prevention of CSA from the very beginning.

## Discussion

Overall, the findings suggest that although there have been significant recent developments in child protection, a number of important issues remain in relation to school-based CSA prevention programs and CSA research in China.

### School-based CSA Prevention Programs

Our findings suggest that participants felt that school-based CSA programs were important. This finding is consistent with a previous study conducted in Australia, in which parents supported these programs even though they did not receive CSA prevention education themselves (Walsh & Brandon, 2012). At the same time, however, some interviewees expressed their concerns about the content of the program and potential negative outcomes (e.g., fear, anxiety) for younger participants. This is in line with previous studies conducted in higher-income countries (HICs) such as Australia and the United States. For example, a qualitative study with 24 parents in Australia suggested that the majority of participants had concerns about potential negative impacts or harms of CSA prevention education (Rudolph & Zimmer-Gembeck, 2018). In another qualitative study exploring teachers’ experiences of implementing a school-based CSA prevention program in the United States, teachers described that some of the parents were concerned about the program content and would opt their children out of the program (Allen et al., 2019).

The finding that interviewees who had delivered CSA programs received inadequate preparation is also consistent with earlier research, which suggests that school teachers are poorly equipped in terms of knowledge about CSA prevention in China (Han & Chen, 2009; Zhang et al., 2015), and highlights the need to empower teachers to deliver CSA prevention programs as an important part of their professional development (Zhang et al., 2015). Similarly, Allen et al.’s (2019) study in the United States revealed that teachers lacked expertise in teaching the CSA prevention program and did not receive adequate training prior to delivering it. Likewise, other studies conducted in the United States and Denmark have suggested that teachers lacked knowledge regarding signs of CSA (Kenny, 2004) and educational resources to teach CSA prevention-related content (Helweg-Larsen et al., 2010; Zeuthen & Hagelskjær, 2013).

Some interviewees in this study doubted the effectiveness of school-based CSA programs in terms of supporting children who have already been abused because of the limited support services for CSA victims in China, and the potential harms that these programs may cause when a child is encouraged to disclose a CSA incident, but no follow-up services are in place. Participants indicated that this is of particular concern in rural areas. In addition, there is no agency primarily responsible for child protection in China, and child protection procedures remain unclear (Man et al., 2017). This has posed challenges for professionals in terms of intervening in child maltreatment cases and providing appropriate support for CSA victims.

### Components of School-based CSA Prevention Programs

There was consensus among participating interviewees that the components of school-based CSA prevention programs identified by the systematic review (Lu et al., 2020) are all essential.

Participants noted the importance of taking cultural factors into consideration when developing school-based CSA prevention programs and proposed a number of additional components that were missing from the original list in order to adapt CSA prevention programs to the Chinese social and cultural context. For example, participants suggested that it is particularly important to utilize a rights-based approach to CSA prevention programs to help restore the power imbalance in the Chinese cultural context, where parents have absolute authority over children and disobedience toward parents is unacceptable (Xu et al., 2019).

In response to the increasing amount of online-facilitated CSA, participants also suggested adding prevention strategies to address this new form of CSA. To date, evidence remains inconclusive with regard to the best strategy to prevent online CSA and school-based prevention programs that target online CSA are primarily implemented in HICs (e.g., *Safer Surfing* in the United Kingdom; *Netsmartz* in the United States; Radford et al., 2014). Evaluation of these programs in HICs has shown promising results in improving children’s understanding of key messages of online CSA (Davidson & Martellozzo, 2008) and there is a clear need for developing context-specific evidence on online CSA prevention strategies in low- and middle-income countries (LMICs).

Last but not least, parental and community involvement were also identified as potential strategies for preventing CSA. Previous studies conducted with parents in mainland China also identified that parents should be engaged in prevention efforts, especially given the lack of government leadership and support services in China (Xie et al., 2016; Chen, Dunne, & Han, 2007).  In terms of community efforts, existing community-based approaches include Barefoot Social Workers, introduced into the Chinese child welfare system in 2010. It has become an important community-based approach to child welfare at the grassroots level (UNICEF, 2018). It should be noted from the interview data that comparing with rural areas where the sense of “community” is stronger, communities in urban areas in China have relatively loose ties among community members. As such, modifications will need to be made to tailor the program to the unique characteristics and needs of urban community members when addressing child protection issues. Furthermore, the effectiveness of the Barefoot Social Workers program still remains unclear and the program has not been associated with CSA prevention directly. Future research should rigorously evaluate this program and assess the potential for integrating CSA prevention components into this existing service.

### CSA Research in China

Participating researchers and professionals discussed the facilitators and barriers to CSA research in China highlighting the way in which an increased awareness of CSA in the central government has fostered research on child protection. Participants also highlighted a lack of national data on violence against children and inadequate government support as major barriers to conducting CSA research. Other barriers that were identified included the need for more effective collaboration across government agencies, a finding that has been highlighted by other research in terms of the need for more coordinated efforts to eradicate CSA in the United States (Wurtele, 2009). Furthermore, professionals and researchers also suggested that more evaluation studies of school-based CSA prevention programs need to be conducted in order to build a best practice knowledge base in the child protection field in China. Only one RCT conducted in China (Lee & Tang, 1998) was included in the most recent systematic review examining the effectiveness of school-based CSA prevention programs (Walsh et al., 2015). Therefore, there is an urgent need to address this evidence gap. In addition, future research should also advocate for policy changes to eliminate the external risk factors for CSA, which involves clarifying the definition of CSA and specifying clear consequences for perpetrators of CSA. Last but not least, efforts need to be made to establish an integrated child protection system that includes early identification, reporting, and response to CSA. At the same time, the system should take the disparities between rural and urban areas into account to improve vulnerable children’s access to child protection services in rural areas.

## Limitations

This study has two major limitations. First, we used snowball sampling to recruit participants from only two sampling locations, which may result in selection bias. However, the participating professionals and researchers in Beijing all had research or practice experiences in different regions of the country (especially rural areas), and as such their views reflect a wide range of experiences across different regions. Second, we did not include children’s perspectives on effective CSA components. As children are the major recipients of school-based CSA prevention programs, their voices should be taken into account when designing future programs or policies. Future studies should examine children’s perspectives on CSA prevention programs and their components. Despite these limitations, our study findings have provided rich insights in terms of the research questions posed and are consistent with and extend findings from previous studies conducted in both China and other countries.

## Implications for Future Policy, Practice, and Research

### Implications for Future Practice and Policy

The findings of this study suggest that while the components of most school-based CSA prevention programs are regarded as being appropriate, some adaptation and development of CSA programs are needed to ensure that they are suited to a Chinese cultural context. The findings also suggest that practitioners believe that school-based programs that do not include the wider community are not likely to be sufficient to address the problem of CSA and as such both parents and the wider community should be involved in CSA prevention efforts. In this regard, the Barefoot Social Worker program could be used to empower community members against CSA by identifying children in need, raising community awareness regarding child protection, and providing training on parenting skills (UNICEF, 2018).

The research also suggests that there is currently inadequate preparation of providers of school-based CSA programs, and inadequate support systems for children who disclose sexual abuse. Better training for those delivering these programs is essential, as is the establishment of an integrated child protection system in China. Furthermore, at a policy and legislation level, this should involve better clarification of the definition of CSA and improved support for CSA victims in China. There is also a need for better collaboration across the different government agencies involved in the delivery of both prevention and treatment services for CSA. Participants underscored the importance of collective engagement of government sectors and ensuring the consistency of child protection practices. To address this, a better-developed child protection system is needed with better coordination in terms of the multiple sectors of government.

### Implications for Future Research

School settings were viewed to be the mainstream platform to deliver CSA prevention curricula but concern about the appropriateness of these programs due to limited support services was voiced. Currently, there is a lack of standardized CSA prevention programs in China and it is imperative that such programs are either developed or adapted from other countries, and that they are then rigorously evaluated to assess their benefits and harms.

Our findings also indicated a need to reduce some of the existing barriers to CSA research implementation and collaboration. This could be addressed in part through the provision of a platform for professionals and researchers to share potential collaboration opportunities and resources. Through the development of a more collaborative working relationship, researchers, professionals, and organizations could potentially improve their capacities, exchange knowledge, and eliminate the gaps between research and practice. Furthermore, previous systematic reviews have indicated that there is a lack of standardized measures to assess any negative effects of these interventions (Topping & Barron, 2009) and therefore investigations of both short- and long- term program effects are needed (Walsh et al., 2015). 

## Conclusion

This is one of the first qualitative studies to examine stakeholders’ perspectives on school-based CSA prevention programs and research in China, and our findings suggest a number of ways in which future CSA prevention programs could be strengthened and adapted to a Chinese context. This study also highlights the importance of breaking down existing barriers to CSA research, including the need for better collaboration, and multi-sectoral and coordinated responses. The findings can also inform the wider development of child protection policies in China.

## Author Biographies

**Mengyao Lu**, MSW, is a DPhil student in Social Intervention at the Department of Social Policy and Intervention, University of Oxford. Her research interest focuses on identifying effective components in school-based child sexual abuse interventions and barriers and facilitators to program implementation in a Chinese context.

**Jane Barlow**, DPhil, is a professor of Evidence-based Intervention and Policy Evaluation at the Department of Social Policy and Intervention, University of Oxford. Jane’s main research interest is the role of early parenting in the aetiology of mental health problems, and the evaluation of interventions aimed at improving parenting practices during pregnancy and the postnatal period. She also undertakes research to evaluate the effectiveness of interventions aimed at preventing child abuse.

**Franziska Meinck**, DPhil, is a lecturer in Social Work at School of Social and Political Science, University of Edinburgh and Honorary Professor at OPTENTIA, Faculty of Health Sciences, North-West University, South Africa. Her research focus is on prevalence, risk, and protective factors of child maltreatment in vulnerable populations in sub-Saharan Africa, health outcomes of violence exposure in childhood, childhood violence prevention and on the development and testing of global child abuse measures.

**Yumeng Wu**, MSPH, is a staff associate at the Aaron Diamond AIDS Research Center at Columbia University Irving Medical Center. Her research focuses on HIV prevention, social-behavioral interventions, and qualitative research. She is interested in using implementation science approach to identify optimal service delivery models for HIV prevention and sexual health services for LGBT population.
